# Growth hormone treatment in the pre-transplant period is associated with superior outcome after pediatric kidney transplantation

**DOI:** 10.1007/s00467-021-05222-5

**Published:** 2021-09-20

**Authors:** Celina Jagodzinski, Sophia Mueller, Rika Kluck, Kerstin Froede, Leo Pavičić, Jutta Gellermann, Dominik Mueller, Uwe Querfeld, Dieter Haffner, Miroslav Zivicnjak

**Affiliations:** 1 Department of Pediatric Kidney, Liver and Metabolic Diseases, Children’s Hospital, Hannover Medical School, Carl-Neuberg-Str. 1, 30625 Hannover, Germany; 2 Zagreb, Croatia; 3 Department of Pediatrics, Division of Gastroenterology, Nephrology and Metabolic Diseases, Charité - Universitätsmedizin Berlin, Campus Virchow-Klinikum, Augstenburger Platz 1, 13353 Berlin, Germany

**Keywords:** Children, Growth hormone, Kidney transplantation, Growth, Kidney function, Development

## Abstract

**Background:**

Recombinant human growth hormone (rhGH) is frequently used for treatment of short stature in children with chronic kidney disease (CKD) prior to kidney transplantation (KT). To what extent this influences growth and transplant function after KT is yet unknown.

**Methods:**

Post-transplant growth (height, sitting height, leg length) and clinical parameters of 146 CKD patients undergoing KT before the age of 8 years, from two German pediatric nephrology centers, were prospectively investigated with a mean follow-up of 5.56 years. Outcome in patients with (rhGH group) and without (non-prior rhGH group) prior rhGH treatment was assessed by the use of linear mixed-effects models.

**Results:**

Patients in the rhGH group spent longer time on dialysis and less frequently underwent living related KT compared to the non-prior rhGH group but showed similar height *z*-scores at the time of KT. After KT, steroid exposure was lower and increments in anthropometric *z*-scores were significantly higher in the rhGH group compared to those in the non-prior rhGH group, although 18% of patients in the latter group were started on rhGH after KT. Non-prior rhGH treatment was associated with a faster decline in transplant function, lower hemoglobin, and higher C-reactive protein levels (CRP). After adjustment for these confounders, growth outcome did statistically differ for sitting height *z*-scores only.

**Conclusions:**

Treatment with rhGH prior to KT was associated with superior growth outcome in prepubertal kidney transplant recipients, which was related to better transplant function, lower CRP, less anemia, lower steroid exposure, and earlier maturation after KT.

**Graphical abstract:**

A higher resolution version of the Graphical abstract is available as Supplementary information

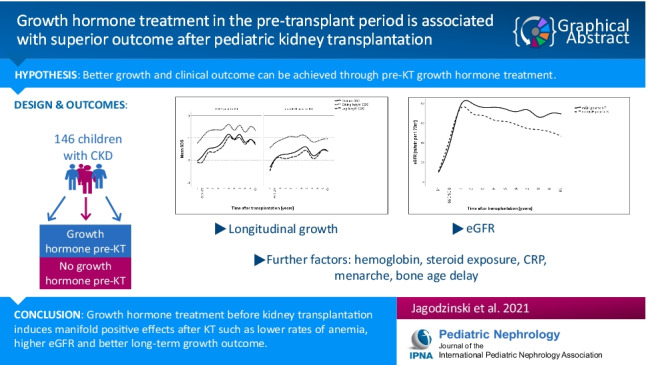

**Supplementary Information:**

The online version contains supplementary material available at 10.1007/s00467-021-05222-5.

## Introduction

Kidney transplantation (KT) is the therapy of choice in pediatric patients with chronic kidney disease (CKD) stage 5 [1]. However, it only manages to correct the disturbances associated with CKD to a certain extent. Indeed, disproportionate short stature with preferential impairment of leg growth is a given in children with CKD stage 5 and catch-up growth after KT is usually limited [2]. Recent studies still report reduced adult height in patients with childhood-onset CKD stage 5 in about 40% of patients despite successful KT [3]. Growth outcome after KT is associated with many factors including patient age, transplant function, steroid exposure, parental height, birth parameters, and initial degree of growth retardation [3–8]. Careful control of caloric intake, metabolic disturbances including acidosis, and the CKD mineral and bone disorder (CKD–MBD), is of utmost importance to achieve optimal height at the time of KT, which is significantly associated with adult height [4, 9]. In addition, treatment with recombinant human growth hormone (rhGH) is a proven measure to improve growth in short children with CKD stages 3–5D [10]. However, rhGH is currently under-utilized in short children with CKD stage 5 in North America and many European countries, which is partly due to family refusal, non-adherence, and lack of reimbursement by insurance companies [11–14]. Its use after KT was also shown to be effective but concern has been raised that it may promote rejection episodes and thereby impair long-term graft function [15]. At present, discontinuation of rhGH therapy at the time of KT is standard practice. The currently recommended strategy for optimizing post-transplant growth consists of steroid-minimizing immunosuppressive protocols and monitoring of spontaneous growth after KT for at least 12 months before considering rhGH treatment [10, 16]. Whether treatment with rhGH prior to KT has long-term effects on growth and clinical features after KT has not been investigated. Therefore, we evaluated post-transplant growth in 146 prepubertal kidney allograft recipients who received rhGH treatment prior to KT or not.

## Methods

### Study design and patients

From May 1998 until January 2020, a total of 947 children who received KT were enrolled in the CKD Growth and Development Study. This is a prospective observational cohort study in two pediatric nephrology centers in Germany (Hannover Medical School and Charité Universitätsmedizin, Berlin) assessing growth and clinical parameters of all available CKD patients stages 3–5D and after KT in yearly intervals as previously described [2]. The study was approved by the local ethics committees, and research was performed in accordance with the Declaration of Helsinki. Study participants and/or their parents gave their consent prior to participation.

For this analysis, all first time graft recipients who underwent KT before the age of 8 years and with at least one valid follow-up examination were included until the age of 18 years. Primary diseases included congenital anomalies of the kidney and urinary tract (59%), glomerulopathies (25%), and others (16%). Patients with syndromic diseases, e.g., Jeune syndrome (*n* = 2), Schimke (*n* = 2), Prune-Belly syndrome (*n* = 1), Arimas syndrome (*n* = 1), and Denys-Drash syndrome (*n* = 1), were excluded. In addition, we excluded patients who had to resume dialysis treatment after KT or who had to be re-transplanted (*n* = 21), who underwent liver or heart transplantations (*n* = 6), or who had to be given rhGH before and after KT (*n* = 9). All annual follow-up interval measurements from immediately after KT (0.01–0.99 years) up to a maximum of 10 (10.00–10.99) years after KT were included. Thus, the data of 146 patients (92 male), who underwent a total of 812 annual measurements, with a mean follow-up of 5.56 years could be analyzed.

Patients were divided in two groups: (i) patients who received rhGH prior to KT (rhGH group, *n* = 52), and (ii) patients who received no rhGH prior to KT (non-prior rhGH group, *n* = 94). In 17 out of 94 patients (18%) of the latter group, rhGH was started after KT, but in none of the former group. Indications for the use of rhGH prior to KT were a height SD score (SDS) <  − 2.0 and a height velocity < 25th percentile in patients with CKD stages 3–5D. The same auxological parameters were used as indications for use of rhGH post KT after a minimum follow-up of 12 months [10].

Primary immunosuppressive protocols included daily prednisolone treatment. By week 8, the prednisolone dosage was tapered down to 4 mg/m^2^/day. All patients were kept on daily prednisolone until 2007. From 2007 onwards, patients were regularly weaned off steroids between six and twelve months post KT in case of stable graft function and lack of rejection. The prescribed dietary intake was in accordance with targeted requirements. Dietary intake was routinely monitored in all CKD and transplanted patients every 3–12 months, depending on patient’s age.

Genetic target height was calculated from mid-parental height: mother’s height + father’s height / 2 ± 6.5 cm in boys and girls, respectively [17]. Data on gestational age were obtained from the children’s health care booklets. The point of attainment of CKD stage 5 was defined by estimated glomerular filtration rate (eGFR) below 15 ml/min/1.73 m^2^, initiation of dialysis, or pre-emptive KT (no prior dialysis). The revised Schwartz formula was used to calculate eGFR [18]. Newborns were classified as small for gestational age (SGA) if birth weight or length was < 10th percentile using national growth charts [19]. Anemia was defined using the World Health Organization age- and sex-specific hemoglobin thresholds for defining anemia in children [20]. There was no significant difference in age distribution of both groups in any year from directly after KT until end of follow-up. Bone age delay was calculated as the difference between bone age and chronological age as a measure of skeletal maturation.

### Anthropometry

Anthropometric measurements included total body height, sitting height, and leg length, as described [21]. The sitting height index was calculated as the ratio between sitting height and stature as a measure of body disproportion. All measurements were performed by the same investigator (M. Z.), as recommended by the International Biological Program [22] with the use of standardized equipment (Dr. Keller, I Stadiometer-Limbach-Oberfrohna, Germany; Siber Hegner Anthropometer Zürich, Switzerland).

### Statistical analyses

Data are given as mean ± SD and/or 95% confidence interval (CI) unless indicated otherwise. SDS values for anthropometric parameters were calculated according to the equation SDS = (*x*_i_–*x*_s_) /SD (*x*_i_ representing individual patient value, *x*_s_ as well as SD representing corresponding value from age- and sex-matched healthy reference peers) [23, 24]. The normality of distribution was evaluated by the Shapiro–Wilk test for each cohort (time after KT) for each variable. Measurements were grouped according to time after KT and age cohorts using yearly intervals. Differences between groups were assessed by unpaired t test or the Mann–Whitney *U* test, as appropriate.

Linear mixed-effects models (Mixed) and the Kronecker product model were used to generate a time-dependent analysis of anthropometric and clinical data including both time after KT and age cohort as well as the factor treatment (rhGH *versus* non-prior rhGH). The combination of unstructured and autoregressive covariance matrix type (UN_AR1) turned out to be the most appropriate for our analyses. In addition, anthropometrical data were adjusted for covariates, i.e., age at CKD stage 5, age at KT, average daily steroid dosage, time after KT, average eGFR, pH, hemoglobin, and HCO_3_ values during the preceding 12 months. Clinical predicted determinants of kidney function after KT in the two treatment groups were adjusted for the following covariates: age at CKD stage 5, age at KT, average daily steroid dosage, time after KT, and averages of hemoglobin, pH, and HCO_3_ during the preceding year. The standard statistical package SPSS for Windows, version 26.0 (IBM Corp), was used for statistical calculations. Results were considered significant at a level of *p* < 0.05.

## Results

**Fig. 1:**
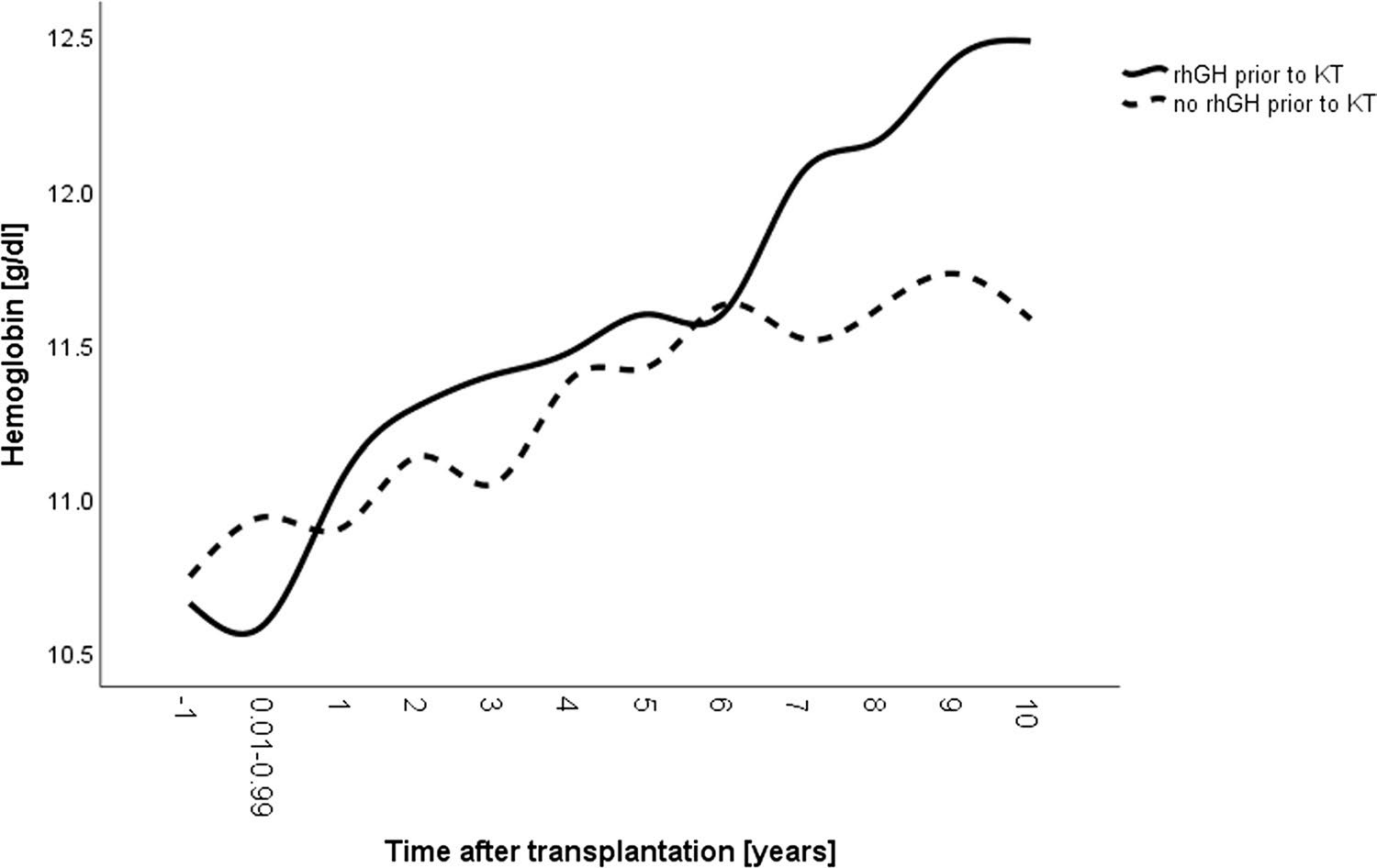
Mean hemoglobin blood concentrations in 146 pediatric kidney transplant recipients with (solid lines, *n* = 52) and without (broken lines, *n* = 94) treatment with recombinant human growth hormone (rhGH) prior to kidney transplantation (KT) during the pre-transplant period. The label − 1 refers to measurements collected in the year prior to KT

Clinical characteristics of the study cohorts are presented in Table 1. Patient groups did not differ with respect to sex, male–female incidence, age when CKD stage 5 was reached and KT, time of follow-up, genetic target height, rates of congenital CKD, pre-emptive KT, and SGA. However, patients in the rhGH group less frequently underwent living related KT (19% versus 34%) and spent longer time on dialysis (median, 1.47 years versus 0.78 years) (Table 1). Growth hormone therapy was initiated in the rhGH group at a median age of 1.93 years, continued over a median period of 1.23 years and was stopped in all patients at the time of KT. By contrast, after KT, rhGH treatment was initiated in the non-prior rhGH group (18%) only. The mean time interval between KT and initiation of rhGH treatment in the patients who received rhGH after KT was 5.71 years. The rhGH group less frequently received treatment with erythropoietin, and less frequently showed anemia which was most pronounced during late post-transplant years (*p* < 0.05) (Table 1, Fig. 1).

**Table. 1:** Clinical characteristics of 146 pediatric kidney transplant recipients with or without rhGH treatment prior to transplantation

	*rhGH prior to KT*			*No rhGH prior to KT*			
	*Incidence*		*No. of cases/measurements*	*Incidence*		*No. of cases/measurements*	*p value*
Male, %	61.5		32 of 52	63.8		60 of 94	0.460
Congenital CKD, %	78.8		41 of 52	77.7		73 of 94	0.522
Preemptive KT, %	34.6		18 of 52	33.0		31 of 94	0.491
Living donor, %	19.2		10 of 52	34.0		32 of 94	0.042
SGA history, %	32.7		16 of 49	24.7		19 of 77	0.220
Epo therapy, %	22.8		71 of 312	28.4		142 of 500	0.044
Iron therapy, %	32.7		102 of 312	35.4		160 of 500	0.448
Vit. D/calcimimetics/phosphate binders, %	41.3		129 of 312	35.7		177 of 500	0.052
Acidosis therapy, %	40.8		125 of 306	40.5		201 of 496	0.493
Anemia, %	41.0		127 of 310	52.1		257 of 493	0.001
*Non-repeated measurements*^*a*^	*Median (IQR)*	*Min.–max*	*No. of cases*	*median (IQR)*	*Min.–max*	*No. of cases*	
Age at KT, years	4.26 (2.78–5.55)	1.35–7.73	52 of 52	3.68 (2.04–5.93)	0.49–7.98	94 of 94	0.529
Duration after KT, years	7.51 (4.23–10.09)	0.06–10.88	52 of 52	8.33 (4.74–10.14)	0.06–10.93	94 of 94	0.608
Age at dialysis initiation, years	2.05 (0.74–4.36)	0.01–7.40	35 of 52	1.92 (0.61–4.33)	0.01–7.81	63 of 94	0.956
Age at CKD stage 5, years	2.94 (1.49–4.90)	0.01–7.40	52 of 52	2.98 (0.94–5.15)	0.01–7.87	94 of 94	0.851
Duration of dialysis, years	1.47 (0.74–2.35)	0.01–4.71	36 of 52	0.78 (0.46–1.66)	0.03–4.75	64 of 94	0.011
Genetic target height, SDS	− 0.10 (–0.67–0.42)	− 2.28–1.46	50 of 52	− 0.13 (–0.67–0.42)	− 2.93–1.39	89 of 94	0.961
Menarche, age	11.79 (11.13–12.24)	10.74–12.97	8 of 20	13.02 (12.80–13.50)	10.21–13.70	7 of 44	0.040
Age at start of rhGH therapy, years	1.93 (1.27–4.13)	0.27–6.31	52 of 52				
Duration of rhGH treatment, years	1.23 (0.59–2.23)	0.11–5.39	52 of 52				
*Repeated measurements*^*b*^	*Estimated marginal mean (95% CI)*	*Min.–max*	*No. of measurements*	*Estimated marginal mean (95% CI)*	*Min.–max*	*No. of measurements*	
eGFR, mL/min per 1.73 m^2*AA*^	71.21 (63.55–78.87)	4.74–147.40	301 of 312	59.20 (53.40–64.99)	8.53–190.54	474 of 500	0.018
Steroid dosage, mg/kg per day^*AA*^	0.04 (0.03–0.05)	0.00–0.33	303 of 312	0.06 (0.05–0.07)	0.00–0.49	476 of 500	0.016
Plasma HCO_3_, mmol/L^*AA*^	22.39 (22.06–22.72)	18.53–31.55	304 of 312	22.72 (22.48–22.97)	18.30–28.32	476 of 500	0.106
Hb, g/dL^*AA*^	11.48 (11.20–11.76)	6.50–14.90	306 of 312	11.39 (11.15–11.63)	7.52–15.53	480 of 500	0.623
PTH, ng/l	89.00 (74.17–103.83)	1.12–641.30	259 of 312	89.80 (79.18–100.43)	5.50–708.00	420 of 500	0.930
CRP, mg/l	2.08 (1.39–2.76)	0.00–50.00	69 of 312	5.59 (2.62–8.57)	0.04–133.95	67 of 500	0.026
Bone age delay, years	–0.71 (− 1.05 to − 0.37)	− 3.34–2.56	130 of 312	− 1.23 (− 1.51 to − 0.94)	− 5.01–4.20	331 of 500	0.023

*IQR* interquartile range, *KT* kidney transplantation, *Epo* erythropoietin, *SGA* small for gestational age, *rhGH* recombinant human growth hormone, *SDS* standard deviation score, *eGFR* estimated glomerular filtration rate, *PTH* parathyroid hormone, *CRP* C-reactive protein

^AA^Annual average: AVERAGE of measurements in the year prior to annual assessment

^a^Descriptive statistic (non-repeated measurements) are given as median and interquartile range (25th–75th percentile)

^b^Repeated measurement (estimated marginal means) during the observation period are based on actual measurement or annual average values (AA), repeated measurements within the same individual (evaluated with the linear mixed model, random patients, and age cohorts)

### Post-transplant growth and maturation in patients with and without prior rhGH treatment

**Fig. 2:**
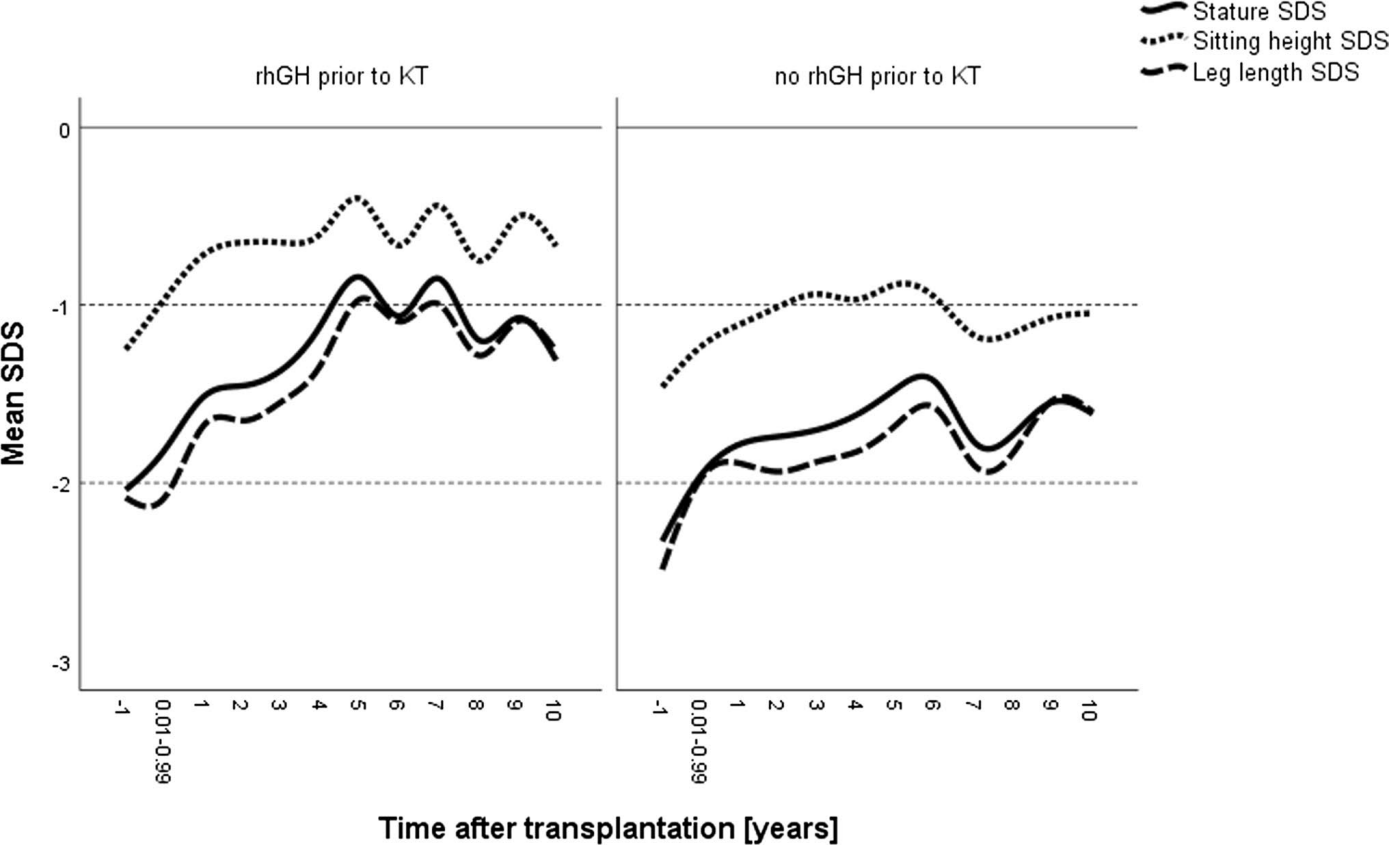
Post-transplant growth in 146 prepubertal children with (left, *n* = 52) and without (right, *n* = 94) treatment with recombinant human growth hormone (rhGH) prior to kidney transplantation (KT). Mean *z*-scores for height, sitting height, and leg length are given. The lower dotted horizontal line refers to the lower normal range (− 2.0 SD score). The label − 1 refers to measurements collected in the year prior to KT

Before KT, *z*-scores for anthropometric data did not differ significantly between groups and 61.3% and 67.9%, respectively, of patients with and without prior rhGH treatment presented with short stature (< − 2.0 SDS) (Fig. 2). After KT, a sustained and significant increase in mean standardized height was noted in patients with prior rhGH treatment (pre-KT, − 2.08 SDS; 4 years post-KT, − 1.11; *p* < 0.05), whereas in patients without prior rhGH treatment the change of standardized height was significant only until 1 year post-KT (Fig. 2). Maximum discrepancy in stature between groups occurred 7 years after KT (− 0.85 SDS versus − 1.76 SDS, *p* < 0.05). Consequently, patients with prior rhGH treatment were generally taller with respect to height, sitting height (each *p* < 0.05), and leg length (*p* = 0.081, Table 2). This was mainly related to a more pronounced increase in leg length in early post-transplant years in the rhGH group, resulting in *z*-scores of − 0.97 in the rhGH group and − 1.67 in the non-prior rhGH group 5 years after KT (*p* < 0.05, Fig. 2).

**Table. 2:** Nonadjusted and adjusted anthropometric parameters in prepubertal kidney allograft recipients who received rhGH before KT or not

	Parameter	Stature SDS	*p* value	Leg length SDS	*p* value	Sitting height SDS	*p* value	Sitting height index SDS	*p* value
*A*	*Non-adjusted*								
	rhGH prior to KT	− 1.33 (− 1.58 to − 1.08)	0.049	− 1.47 (− 1.72 to − 1.22)	0.081	− 0.71 (− 0.95 to − 0.47)	0.026	1.20 (0.92 to 1.47)	0.937
	No rhGH prior to KT	− 1.68 (− 1.92 to − 1.43)		− 1.79 (− 2.04 to − 1.53)		− 1.08 (− 1.31 to − 0.85)		1.18 (0.95 to 1.41)	
*B*	*Adjusted*								
	rhGH prior to KT	− 1.34 (− 1.65 to − 1.03)	0.136	− 1.49 (− 1.76 to − 1.22)	0.154	− 0.67 (− 0.92 to − 0.41)	0.046	1.21(0.92 to 1.49)	0.849
	No rhGH prior to KT	− 1.64 (− 1.90 to − 1.39)		− 1.78 (− 2.08 to − 1.49)		− 1.03 (− 1.28 to − 0.78)		1.24 (0.98 to 1.51)	

A: Non-adjusted—Data are presented as SD scores (SDS), estimated marginal means (95% confidence intervals). *p* values are based on the linearly independent pairwise comparisons among the estimated marginal means

B: Adjusted—Data are presented as estimated marginal means (95% confidence intervals); age at chronic kidney disease stage 5, age at KT, average daily steroid dosage in mg per kg per analyzed year, time after KT, pH values, hemoglobin average of last year, HC0_3_ average of last year and eGFR; *p* values are based on the linearly independent pairwise comparisons among the estimated marginal means

*SDS* standard deviation score, *KT* kidney transplantation, *rhGH* recombinant human growth hormone, *eGFR* estimated glomerular filtration rate

Consequently, the frequency of short stature after KT was lower in the rhGH group (35.7% versus 50.0%) which was most pronounced 7 years after KT (11.1% versus 45.2%, *p* < 0.01). Likewise, reduced sitting height and leg length (< − 2.0 SDS) were more frequently noted in the non-prior rhGH group which was also most pronounced 7 years after KT (sitting height, 24.4% versus 0.0%; leg length, 48.8 versus 11.1%; each *p* < 0.05). By contrast, the mean standardized sitting height index was comparably elevated in both groups (Table 2).

The typical prepubertal peak of growth in stature and leg length occurred 5 years after KT in the rhGH group, whereas the non-prior rhGH group showed a delay in leg length and total body height, peaking 6 years after KT (Fig. 2). Instead, sitting height showed the same timing in growth gain in both groups, peaking 5 years after KT, but differing in growth intensity (rhGH group − 0.41 versus non-prior rhGH group − 0.92 SDS).

**Fig. 3:**
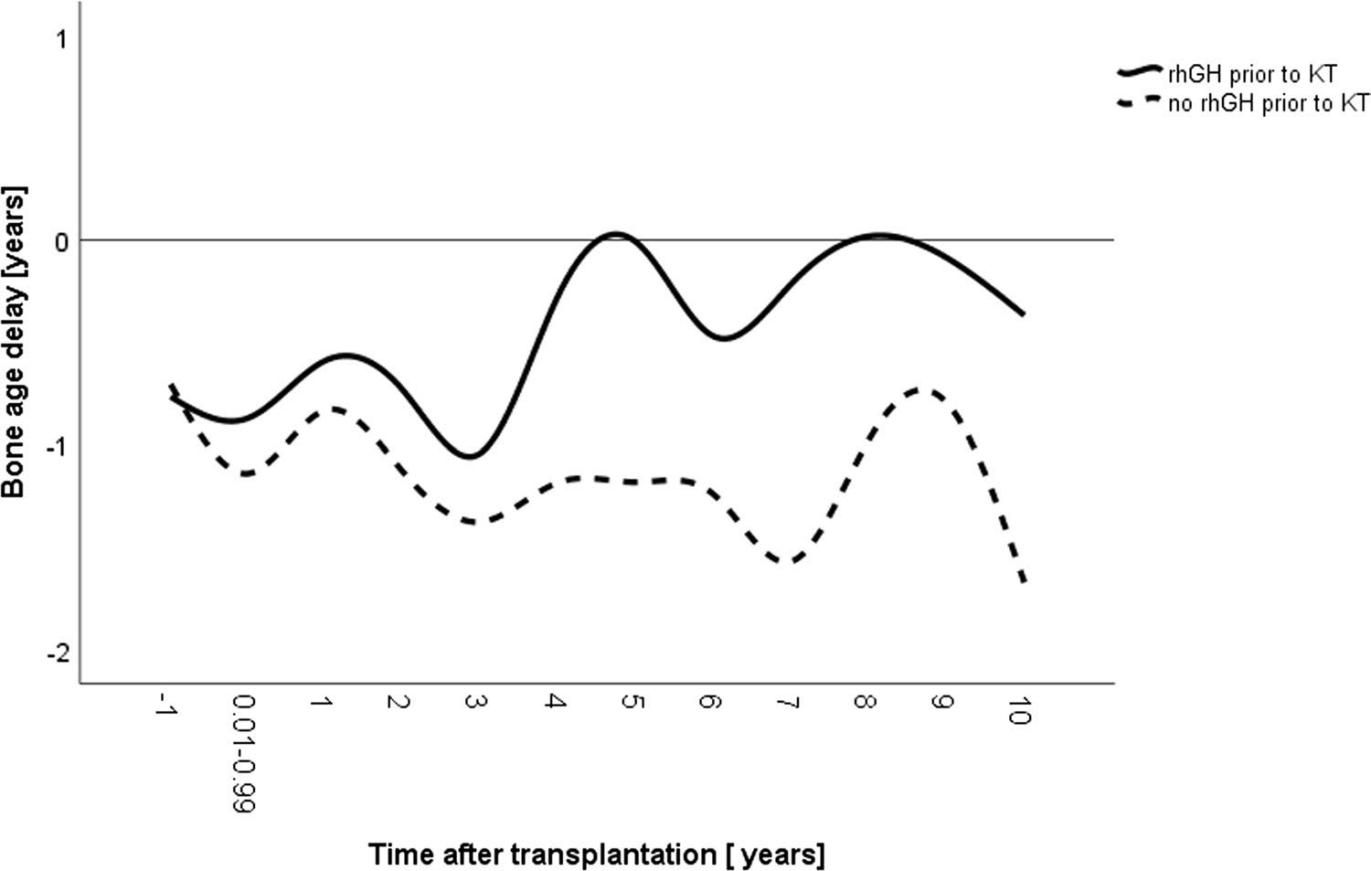
Mean bone age delay in 146 pediatric kidney transplant recipients with (solid lines, *n* = 52) and without (broken lines, *n* = 94) treatment with recombinant human growth hormone (rhGH) prior to kidney transplantation (KT). The label − 1 refers to measurements collected in the year prior to KT

Likewise, age at menarche occurred much later in the non-prior rhGH group than in the rhGH group (13.02 years versus 11.79 years, respectively, *p* < 0.05, Table 1). Mean bone age delay was comparable in both groups at last assessment before KT (*p* = 0.497), but was significantly more pronounced in the non-prior rhGH group after KT (rhGH group − 0.71 years; non-prior rhGH group − 1.23 years, *p* < 0.05, Table 1, Fig. 3).

### Transplant function and biochemical parameters in patients with and without prior rhGH treatment

**Fig. 4:**
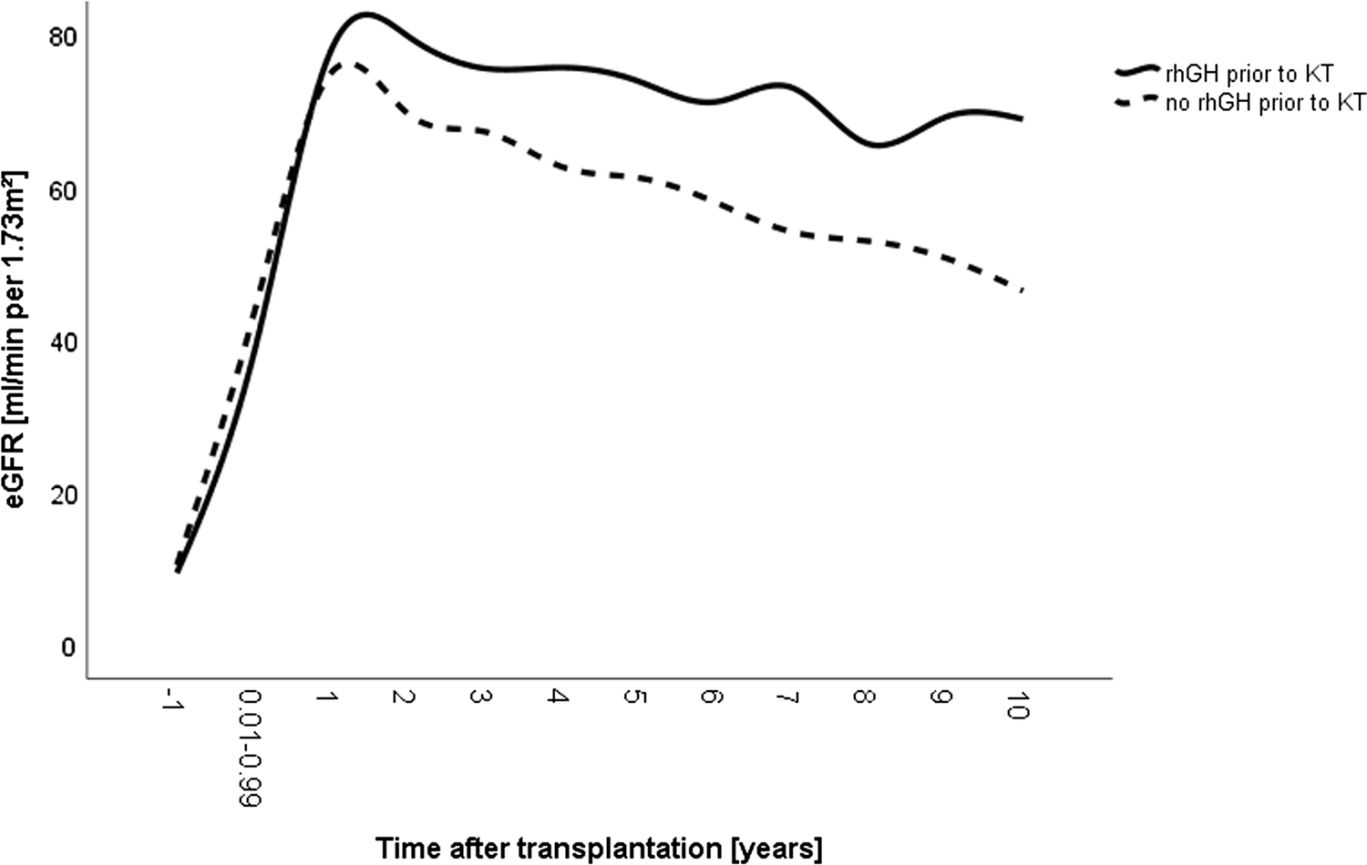
Mean estimated glomerular filtration rates in 146 pediatric kidney transplant recipients with (solid lines, *n* = 52) and without (broken lines, *n* = 94) treatment with recombinant human growth hormone (rhGH) during the pre-transplant period. The label − 1 refers to measurements collected in the year prior to KT. KT, kidney transplantation, eGFR, estimated glomerular filtration rate

**Fig. 5:**
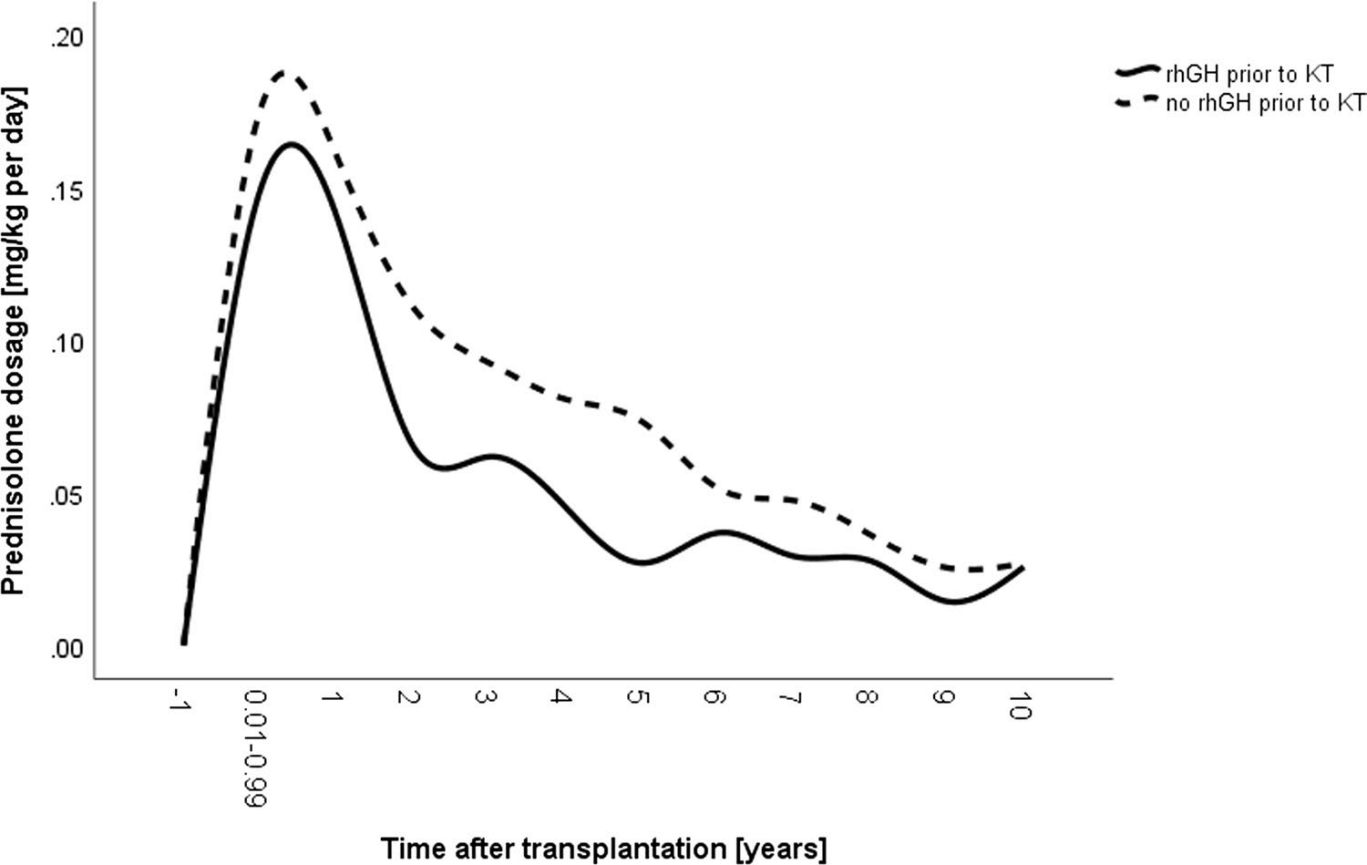
Mean daily prednisolone dosages in 146 pediatric kidney transplant recipients with (solid lines, *n* = 52) and without (broken lines, *n* = 94) treatment with recombinant human growth hormone (rhGH) prior to kidney transplantation (KT). The label − 1 refers to measurements collected in the year prior to KT

Mean eGFR values were significantly higher in the rhGH group after KT overall (71.21 ml/min/1.73 m^2^ versus 59.20 ml/min/1.73 m^2^) (Table 1). Mean eGFR peaked 1 year and 2 years post-KT and amounted to 75 ml/min/1.73 m^2^ and 80 ml/min/1.73 m^2^ in the non-prior rhGH group and the rhGH group, respectively (Fig. 4). Significant decrease of mean eGFR began in the non-prior rhGH group 4 years post-KT, whereas in the rhGH group it began 8 years after KT (each *p* < 0.01) resulting in a substantially higher eGFR 10 years after KT in the latter group (69 ml/min/1.73 m^2^ versus 46 ml/min/1.73 m^2^, *p* < 0.01) (Fig. 4). In both groups, post-transplant eGFR was associated with time after KT, steroid dosage, and circulating hemoglobin levels (each *p* < 0.01, Table 3), whereas bicarbonate levels were positively associated with post-transplant eGFR only in the non-prior rhGH group (*p* < 0.05). The rhGH group had lower steroid exposure than those without prior rhGH treatment (*p* < 0.05) (Table 1, Fig. 5). Likewise, mean C-reactive protein (CRP) levels were lower in the rhGH group (2.08 mg/l versus 5.59 mg/l, *p* < 0.05). By contrast, mean PTH and plasma HCO_3_ levels were comparable in both groups (Table 1).

**Table. 3:** Clinical predictors of transplant function

Parameter	eGFR: rhGH prior to KT	*p* value	eGFR: No rhGH prior to KT	*p* value
Age at stage 5 CKD (in year)	0.11 (− 4.56 to 4.79)	0.961	4.37 (− 3.99 to 12.72)	0.301
Age at KT (in years)	− 1.59 (− 7.14 to 3.96)	0.567	− 5.90 (− 15.16 to 3.35)	0.209
Time after KT (in years)	− 2.96 (− 3.69 to − 2.23)	0.000	− 2.39 (− 2.75 to − 2.02)	0.000
Steroid dosage (in mg/kg)	− 99.14 (− 133.20 to − 65.09)	0.000	− 60.92 (− 87.03 to − 34.81)	0.000
Hemoglobin (in 10* g/dl)^AA^	4.27 (2.79 to 5.75)	0.000	1.84 (0.77 to 2.92)	0.001
Plasma HCO_3_ (in 10*mmol/l)^AA^	0.03 (− 1.08 to 1.14)	0.957	0.85 (0.11 to 1.59)	0.024

*eGFR* estimated glomerular filtration rate, *KT* kidney transplantation, *CKD* chronic kidney disease

^AA^Annual average

### Adjusted anthropometric z-scores in patients with and without prior rhGH treatment

After adjusting anthropometric data for potential confounders (including age at which CKD stage 5 was reached, age at KT, average daily steroid dosage, pH value, time after KT, and average eGFR, bicarbonate, and hemoglobin levels during the preceding year), significant differences between groups were limited to sitting height *z*-scores (Table 2).

## Discussion

This study shows that treatment of children with rhGH in the pre-transplant period was associated with several long-term beneficial effects after transplantation, some of which were unexpected. rhGH not only improved growth and maturation after KT but was also associated with better long-term transplant function, and lower degree of anemia and inflammation. Our data confirm the limited potential for substantial catch-up growth after KT and support the concept of timely initiation of rhGH treatment prior to KT in children with CKD and persisting short stature.

Growth failure is a hallmark of pediatric CKD, especially CKD stage 5 [3, 25], as also exhibited in this young CKD stage 5 population with mostly congenital CKD in which short stature was noted in approximately 2/3 of patients. In about 35% of patients, treatment with rhGH was commenced prior to transplantation due to persistent short stature as recommended by current guidelines [10, 26]. Consequently, mean height *z*-scores at the time of KT did not differ between children with or without prior rhGH treatment. After KT, rhGH treatment was initiated in 18% of patients without prior rhGH treatment but in none of the rhGH group. Nevertheless, post-transplant growth was superior in the latter group. This is even more remarkable since these patients, who spent longer time on dialysis and less frequently underwent living related KT were burdened with conditions associated with poor growth outcome [27, 28].

The prospective assessment of post-transplant changes in linear body dimensions of both groups displayed disproportionate short stature with predominant impairment of leg length and rather preserved trunk length, which is in line with previous studies [21]. This is known to result in a significantly elevated sitting height index compared to healthy children [2]. After KT, superior height gain in the prior rhGH group occurred after an initial better preservation of sitting height followed by an improved long-term catch-up growth with increasing leg length. We previously demonstrated that KT preferentially stimulates trunk growth in young children (age < 4 years) and leg growth in older children resulting in harmonization of body proportions [8]. This reversible variation in the sizes of body segments known as *phenotypic plasticity* seems beneficial for the organism in adapting to changes in living conditions or illness [29]. The present study suggests that pre-treatment with rhGH may prime the body to undergo phenotypic plasticity after KT and thereby helps to harmonize body proportions in these patients. As a consequence, the percentage of patients with normal height after KT was substantially higher in the rhGH group compared to that in the non-prior rhGH group which was most pronounced at 7 years after KT (88.9% versus 54.3%) corresponding to a mean age of 12 years.

With the typical prepubertal peak of linear growth occurring 1 year later in the non-prior rhGH group, our results suggest delayed onset of puberty and/or reduced pubertal height gain in these patients. Similar differences were observed with respect to sexual maturation. Menarche occurred timely in the rhGH group but was delayed by approximately 1 year in the non-prior rhGH group. Previous studies showed that longer duration of CKD, corticosteroid use, and lower GFR as well as shorter stature were associated with delayed menarche [30], and that girls with delayed menarche had lower bone mass density [31], which is in line with our results. Taken together, our data suggest that pre-treatment with rhGH improves growth as well as sexual and skeletal maturation after KT.

How does pre-treatment with rhGH impact on post-transplant growth and maturation? Evidence in this regard comes from two prospective studies investigating the effects of rhGH treatment on bone histology and matrix mineralization in short children on dialysis [32, 33]. Both studies showed that rhGH not only improved growth in these patients, but also normalized bone formation rates as well as bone matrix mineralization irrespective of the type of histologic feature, e.g., abnormal bone turnover, mineralization, and/or volume, whereas no such changes were noted in controls. Therefore, pre-treatment with rhGH also might have improved bone quality in the present study and thereby prepared the bone for optimal post-transplant growth compared to patients without prior rhGH treatment.

Other important factors known to affect post-transplant growth including allograft function and steroid exposure need to be considered as well. Long-term transplant function in pediatric kidney allograft recipients is influenced by the quality of the transplant itself, with better results with living related donors, recipient-related factors such as HLA-matching and HLA-immunization and the immunosuppressive regimen [1, 34, 35]. In the present study, the frequency of living related KT was lower in the rhGH group compared to the non-prior rhGH group. This is most likely due to the fact that patients for whom living-related transplantation is not an option in the near future are known to spend longer time on dialysis treatment [36], further exacerbating growth impairment. In those cases, physicians and families are more likely to initiate rhGH treatment. Despite this, long-term transplant function was superior in the rhGH group compared to that in the non-prior rhGH group. Although, in this observational study the primary immunosuppressive regimens did not generally differ between patients with or without prior rhGH treatment, the latter group received a substantially higher steroid exposure, which may at least partly explain the inferior growth outcome in this group. Lower steroid exposure in the rhGH group may be the consequence of better graft function facilitating steroid minimizing/withdrawal in these patients.

Elevated CRP levels were noted in the non-prior rhGH group but not in the rhGH group. C-reactive protein is a marker of the acute phase response to inflammation. Elevated circulating CRP (> 3 mg/L) is associated with accelerated deterioration of graft function in kidney transplant recipients and thought to reflect kidney inflammation due to subclinical rejection [37–41]. Therefore, the notion of significantly elevated CRP levels in the non-prior rhGH group may not only explain the higher frequency of anemia as a consequence of inflammation but also subclinical graft rejection in these patients which may contribute to the inferior long-term graft function.

Finally, growth hormone and its mediator insulin-like growth factor (IGF) 1 play an important role in immunoregulation. Immune cells such as T and B lymphocytes express GH and IGF receptors and are therefore targets of both GH and IGF1 which may act as local growth and differentiation factors [42] and may regulate cytokine reaction [43]. However, a meta-analysis found no elevated rejection rates or more rapid deterioration of graft function due to rhGH treatment in pediatric kidney allograft recipients [44]. Data on the immunomodulatory effects of rhGH in pediatric CKD patients prior to KT are lacking. In view of the superior graft function in patients with prior rhGH treatment in the present study, it is tempting to speculate that pre-treatment with rhGH might have modified the immune response to the kidney allograft positively. However, several potentially confounding factors such as HLA mismatch, HLA antibodies, non-steroid immunosuppressive therapies, and allograft biopsy results could not be addressed in our study. An analysis with inclusion of patients who received rhGH before, as well as after KT was performed in which there was no marked difference to the analysis which excluded those patients, possibly due to the small number of those patients (*n* = 9). Therefore, a dedicated analysis regarding impact of and reasons for rhGH treatment in patients before, as well as after transplantation, is an interesting subject for further research.

In conclusion, pre-KT rhGH treatment in pediatric kidney allograft recipients resulted in superior long-term growth outcome after KT compared to patients without prior rhGH treatment which was mainly due to improved leg growth as well as skeletal maturation. Our data suggest that treatment with rhGH in the pre-transplant period in CKD patients presenting with persistent short stature is not only useful to improve growth outcome, but rather induces manifold positive effects such as lower rates of inflammation and anemia as well as better preservation of transplant function.

## Supplementary Information

ESM 1 (PPTX 85.2 KB)

## Data Availability

Data cannot be published as it is used in an ongoing study.
