# Supplementary material for: Growth hormone treatment in the pre-transplant period is associated with superior outcome after pediatric kidney transplantation
Source: Pediatr Nephrol. 2021 Sep 20;37(4):859–69. doi: 10.1007/s00467-021-05222-5 (PMC8960657; doi:10.1007/s00467-021-05222-5)
Supplement: Supplementary file 1 — (PPTX 85.2 KB) [file 467_2021_5222_MOESM1_ESM.pptx]

## Slide 1
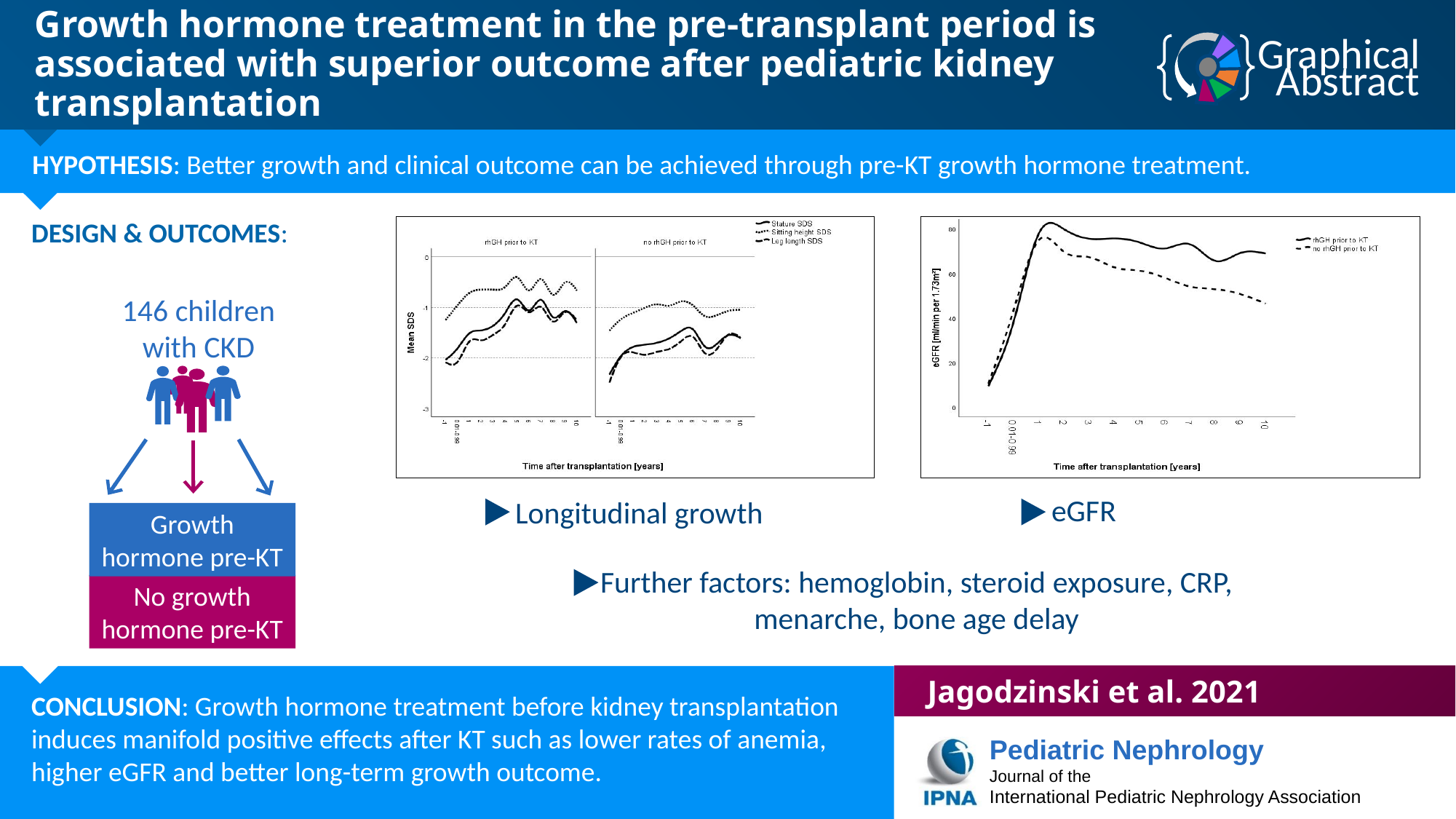

Growth hormone treatment in the pre-transplant period is associated with superior outcome after pediatric kidney transplantation
HYPOTHESIS: Better growth and clinical outcome can be achieved through pre-KT growth hormone treatment.
DESIGN & OUTCOMES:
146 children with CKD
eGFR
Longitudinal growth
Growth hormone pre-KT
Further factors: hemoglobin, steroid exposure, CRP, menarche, bone age delay
No growth hormone pre-KT
Jagodzinski et al. 2021
CONCLUSION: Growth hormone treatment before kidney transplantation induces manifold positive effects after KT such as lower rates of anemia, higher eGFR and better long-term growth outcome.
